# Apraxia Patterns for the Differentiation between Alzheimer’s Disease and Frontotemporal Dementia Variants

**DOI:** 10.3390/medicina60030435

**Published:** 2024-03-06

**Authors:** Georgios Papadopoulos, Dimitrios Parissis, Anna Gotzamani-Psarrakou, Panagiotis Ioannidis

**Affiliations:** 1 2nd Department of Neurology, AHEPA University Hospital of Thessaloniki, 54636 Thessaloniki, Greece; 2 Department of Neurology, 401 Military Hospital of Athens, 11525 Athens, Greece; 3 Laboratory of Nuclear Medicine, AHEPA University Hospital of Thessaloniki, 54636 Thessaloniki, Greece

**Keywords:** apraxia, frontotemporal dementia, Alzheimer’s disease

## Abstract

*Background and Objectives*: Despite the increasing use of biomarkers, differentiation between Alzheimer’s disease (AD), behavioral variant Frontotemporal Dementia (bvFTD), and Primary Progressive Aphasia (PPA) remains a challenge. Apraxia is a supportive feature for diagnosing AD but is underrepresented in other dementia types. Herein, we investigated the presence and characteristic profiles of limb, verbal, and non-verbal apraxia in three major dementia types. *Materials and Methods*: Test for Upper Limb Apraxia (TULIA) and Apraxia Battery for Adults—2 (ABA-2) were administered in patients with AD (n = 22), bvFTD (n = 41), and PPA (n = 22), with 20 individuals serving as healthy controls (HC). Composite and subdomain scores were compared between each patient group and the HC. Praxis profiles indicative of each dementia type and a possible predictive value were sought. *Results*: Apraxia provided high diagnostic accuracy for detecting dementia compared with HC (sensitivity: 63.6–100%, specificity: 79.2–100%). Patients with AD performed worse when imitating intransitive gestures as well as pantomiming transitive gestures (mean differences: 2.10 and 3.12, respectively), compared with bvFTD. PPA patients, compared with bvFTD, had comparable results in limb, verbal, and non-verbal praxis assessments, despite the greater deterioration in the outcome. Compared with patients with AD, PPA had increased pathological outcomes in verbal (86.4% vs. 40.9%) and non-verbal apraxia (31.8% vs. 0%), while bvFTD had increased pathological outcomes in verbal apraxia (85.4% vs. 44.5%). Finally, apraxia is correlated with cognitive decline. *Conclusions*: Apraxia profile evaluation could contribute to the differentiation between AD and Frontotemporal Dementia (FTD). Both TULIA and ABA-2 are reliable tools that can be performed as bed-side tests in clinical practice.

## 1. Introduction

Maintaining the quality of life of patients with dementia is the main priority of the treating physician. Despite the increasing role of cerebrospinal fluid (CSF) biomarkers in the diagnosis of Alzheimer’s disease and the increasing validity of plasma biomarkers, the criteria remain largely clinical. Apraxia is one of the main clinical findings not receiving the required attention and is currently the subject of simple observation. Although it is known that patients with Alzheimer’s disease (AD) and non-fluent variant primary progressive aphasia (nfvPPA) will develop ideomotor and verbal apraxia, respectively [1,2], the manifestation of the full spectrum of praxis disturbance in frontotemporal dementia (FTD) and AD has not been investigated.

Apraxia is the inability to achieve learned or skilled movements, which is not due to motor deficits, sensory disorders, or language comprehension disorders [3]. Ιdeomotor apraxia is defined as a disorder in the execution of meaningful or meaningless gestures. According to literature, three systems are involved in the praxis circuit: the perceptual (processing visual and auditory stimulus), the conceptual (semantic knowledge and conceptual depiction), and the production system (extracting the concept of a movement into a sequence of actions) [4]. Disturbances in praxis are mainly the result of frontal and parietal lesions in the left hemisphere, although visual and auditory inputs are also involved (temporal and occipital participation). The same anatomical and functional structures are involved in verbal and non-verbal apraxia. These disturbances arise from motor programming deficits at the level of speech mechanisms and oral muscles, as well as comprehension of emotional and cognitive states (social cognition) [5]. Added to ideomotor apraxia, these “face apraxias” constitute the core expressions of praxis circuit lesions.

The purpose of this study is threefold. Initially, ideomotor, verbal, and non-verbal apraxia was recorded and studied in patients with AD and FTD using the Test for Upper Limb Apraxia (TULIA), a diagnostic tool that evaluates the conceptual and production system of the praxis circuit in the upper extremities, and the Apraxia Battery for Adults-2 (ABA-2), which was recently modified and validated in the Greek population with dementia and serves for the examination, mainly, of apractic findings in speech and buccofacial area, and to a lesser degree of limb apraxia. Our second goal was to compare the findings between patients with dementia and healthy controls (HC), to explore the significance of apraxia in the clinical setting of these dementia categories, and to correlate praxis disturbances with cognitive impairment. Finally, an attempt was made to define the types of apraxia seen in patients with AD and FTD that could be used in the differential diagnosis process. Thus, a specific rehabilitation program targeting the exact disability that our patient is expected to face based on the diagnosis could be launched.

## 2. Materials and Methods

### 2.1. Participants

All patients diagnosed with AD, bvFTD, and PPA at the 2nd Department of Neurology of the AHEPA University Hospital of Thessaloniki, Greece, between January 2014 and March 2018 were included in the study. Diagnosis was made by a team of trained clinicians according to current clinical criteria [1,2,6]. All patients with AD (n = 22) presented with early memory impairments that worsened progressively, whereas patients with bvFTD (n = 41) showed personality changes and social conduct disturbances with relatively sparing memory, and patients with PPA (n = 22) showed speech production, object knowledge, and naming disturbances. Individuals were already diagnosed with dementia and were on cholinesterase inhibitors (donepezil or rivastigmine) or were first diagnosed during enrollment.

Medical history as well as behavioral, memory, and language disturbances were confirmed by relatives or caregivers. All patients were native Greek speakers who met inclusion and exclusion criteria. Specifically, patients with a history of major mental illness, head trauma, epilepsy, alcoholism, or medication-causing motor dysfunction were excluded from the study. Magnetic resonance imaging (MRI) was used to exclude stroke, severe cerebrovascular disorder, brain lesion, or hydrocephalus for each individual, as well as to confirm findings of focal atrophy consistent with the clinical diagnosis. To confirm the diagnosis of dementia, the Adenbrooke’s Cognitive Examination–Revised (ACE-R) battery, modified and validated in the Greek population, was used [7]. Frontal Behavioral Inventory (FBI) and Frontal Rating Scale (FRS) batteries were followed to differentiate FTD from the AD population and to distinguish individuals with moderate or severe functional disorders [8,9]. Apraxia was tested with TULIA and ABA-2. Finally, single photon emission computed tomography (SPECT) was conducted on all patients and used as a biomarker of hypoperfusion and, indirectly, neural damage, strengthening the diagnosis made based on the existing criteria [10,11].

A control group of 20 healthy volunteers was also included. Their age, gender, and education were matched to our patient sample. This group consisted mainly of patient attendants in order to avoid bias due to demographic characteristics. Clinical examination and neuropsychological assessment prior to enrollment confirmed they fulfill the inclusion and exclusion criteria of the study. An MRI scan was not considered necessary in this study group.

All participants were examined in a quiet and comfortable room. Signed informed consent was obtained by all participants (or caregivers). This study was approved by the local ethical committee, and the principles of the Helsinki Declaration were followed. STARD reporting guidelines checklist was used [12]. The authors declare that they have no known competing financial interests or personal relationships that could have appeared to influence the work reported in this paper.

### 2.2. Apraxia Assessment

Test for Upper Limb Apraxia (TULIA) is a reliable and valid bedside test for evaluating gesture production. Imitation and pantomime tasks are included that are tool-related (transitive), communicative (intransitive), and non-symbolic (meaningless). Imitating gestures requires an intact “production system” (frontal areas) with the ideational level being overtaken, while, in pantomime, parietal areas are, firstly, activated to recreate the correct sequence of movements (conceptual system), and then frontal areas extract the concept of motion into a sequence of movements. Transitive, intransitive, and meaningless gestures examine simple as well as demanding actions that rely either on stored knowledge or are independent of any usage in the past. Each subtest consists of 8 items with a scoring method ranging from 0 (no movement—unrecognizable movement) to 5 (normal movement—identical to the demonstrated movement) (total score range 0–240) [13].

Apraxia Battery for Adults—2 (ABA-2) consists of 8 subtests that detect, mainly, verbal (5 subtests) and non-verbal (1 subtest) apraxia and roughly limb apraxia (1 subtest). Verbal apraxia (or apraxia of speech) relies on the left frontal lobe (specifically posterior inferior areas) and anterior insula area, while atrophy of the prefrontal cortex and supplementary motor area are associated with non-verbal (or buccofacial) apraxia. In ABA-2, patients are categorized by the presence, the degree, and the type of apraxia, and the results are used for the guidance of the therapeutic approach. Recently, we published the validation for this battery in Greek patients with dementia and have suggested a modified version with equal diagnostic value; this version was administered to all participants of our study [14,15]. 

### 2.3. Statistical Analysis

Statistical analyses were performed using IBM SPSS Statistics for Windows, version 25.0 (IBM Corp., Armonk, NY, USA). Continuous data are presented as mean ± standard deviation (SD) or median (Q25–Q75) and categorical as absolute and relative frequency. Missing data were below 1% for all variables. The normality of the distribution of data was examined using the Kolmogorov-Smirnov test and Q-Q plots. Pearson’s chi-square test (or Fisher’s exact test) followed by multiple comparisons using the Bonferroni method was used to compare categorical variables among the groups. Continuous variables were compared among the groups using analysis of variance (ANOVA) or the Kruskal–Wallis test, followed by Bonferroni correction or the Dunett post hoc test. Receiver operating characteristic (ROC) curves were generated, and the area under curve (AUC) was calculated to determine the diagnostic performance of the apraxia batteries. Spearman’s rho was used to determine correlations between continuous variables. Multivariable linear and binary logistic regression analyses (including age, sex, years of education, and disease duration) were conducted to explore the association between ACE-R scores and apraxia battery outcomes, as well as to evaluate the diagnostic value of subdomain scores. In multivariable linear regression analyses, only results with R^2^ > 0.2, B > 0.1 or <−0.1, and *p* < 0.5 are presented. A *p*-value ≤ 0.05 was considered statistically significant.

## 3. Results

Socio-demographic characteristics, age, gender, and education level, as well as disease duration, did not differ amongst HC and patients with AD, bvFTD and PPA. FBI and FRS confirmed the patient’s diagnosis and played a role in staging the disease’s severity. Patients with bvFTD presented with higher FBI scores compared with AD and PPA groups, while patients with PPA did not pass the expected cut-off values but scored significantly higher than AD. Results from ACE-R did not differ between AD and bvFTD groups; however, patients with PPA presented with lower scores.

**Figure 1:**
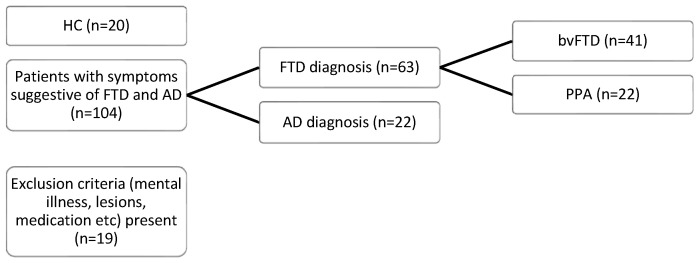
Flow chart illustrating selection of participants. HC: Health Control, AD: Alzheimer’s Disease, FTD: Frontotemporal Dementia, bvFTD: behavioral variant FTD, PPA: Primary Progressive Aphasia, TULIA: Test for Upper Limb Apraxia, ABA-2: Apraxia Battery for Adults—2.

A schematic illustration of patient selection is presented in Figure 1.

### 3.1. Composite Scores

All patients scored significantly lower than the HC when examined with the TULIA and ABA-2 batteries. More specifically, regarding TULIA, patients with PPA had the lowest scores compared with other patient categories, followed by patients with AD (Table 1). 

We compared composite scores of TULIA between each dementia group and HC and found that differences were statistically significant in all comparisons. However, TULIA scores did not show statistically significant differences between the FTD vs. AD, PPA vs. AD, bvFTD vs. AD, and bvFTD vs. PPA groups. ABA-2 administration showed that the majority of patients with AD and FTD (both bvFTD and PPA) had scores indicating limb, verbal, and non-verbal apraxia (63.6%, 90.2%, and 90.9% of patients, respectively). Contrariwise, only 4.2% of the HC group had abnormal findings. The results of composite scores differed when comparing all patient categories with HC (*p* < 0.001 for all group comparisons). Performance significantly differed between FTD vs. AD and bvFTD vs. AD individuals (worse results for patients with FTD), while no difference was found between bvFTD vs. PPA groups and PPA vs. AD groups (Table 2).

**Figure 2:**
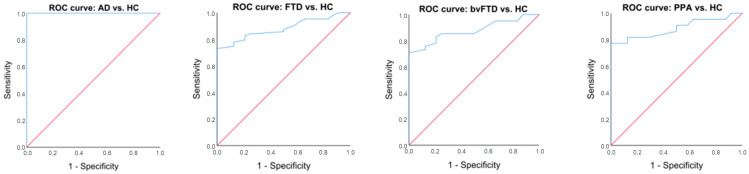
Roc Curve analysis comparing AD vs. HC, FTD vs. HC, bvFTD vs. HC, PPA vs. HC.

Overall, predicting the presence of dementia, as well as its specific type, versus HC was possible using the composite score of both screening tests. The highest diagnostic accuracy (AUC = 1.00) of TULIA was observed for AD vs. HC, while the remaining groups also demonstrated sufficient diagnostic accuracy (AUC > 0.87). The sensitivity and specificity of TULIA were sufficient for all dementia groups, especially the AD group, as was ABA-2 for the FTD groups. Based on the AUC analysis, new cutoff values are suggested to discriminate each dementia group from HC to achieve optimal sensitivity and specificity rates (Table 3, Figure 2).

Furthermore, the correlation between apraxia and the ACE-R score was examined to determine the effect of global impairment in praxis. The decrease of composite scores in limb praxis, as measured with TULIA, affected moderately cognitive functions in patients with AD, FTD, and bvFTD (r = 0.632, *p* = 0.002; r = 0.594, *p* < 0.001; r = 0.467, *p* = 0.002, respectively) and strongly in the PPA group as well (r = 0.747, *p* < 0.001), according to Spearman’s rho coefficient. Multivariate linear regression revealed comparable results only for the FTD and bvFTD groups (R^2^ = 0.405, B = 1.032, *p* < 0.001; R^2^ = 0.278, B = 0.610, *p* = 0.007, respectively).

Moreover, multivariate binary logistic regression was used to examine the correlation between the ABA-2 outcome and the ACE-R score. In the total population, ACE-R was a negative independent predictor (B = −0.053, SE = 0.022, *p* = 0.018) of the probability of pathological outcome, with the odds ratio (OR) indicating that for every one unit increase on this predictor, the odds of pathological outcome change by a factor of 0.948 (95% CI: 0.907–0.991). In the FTD group, ACE-R was a negative independent predictor (B = −0.78, SE = 0.039, *p* < 0.044, OR = 0.925, 95% CI: 0.857–0.988). In AD patients as well as in bvFTD and PPA groups, ACE-R was not found to be a significant predictor of the ABA-2 outcome.

### 3.2. Apraxia Subdomains

A detailed analysis of each praxis task for the four groups of patients showed similarities but also distinct findings, depending on the diagnosis. Compared with HC, all four groups showed significantly reduced scores in all subdomains of TULIA and an increased percentage of pathological outcome in ABA-2, except subtest 4 (“Latency Time and Utterance Time for Polysyllabic Words”—evaluation of apraxia of speech) for all dementia groups vs. HC comparisons and subtests 3A and 3B (“limb apraxia” and “buccofacial apraxia”) for HC vs. bvFTD (Table 4).

Between-group comparisons revealed distinct limb apraxia patterns for each dementia group, according to the TULIA battery. bvFTD vs. AD showed statistically significant differences in imitation of intransitive gestures, as well as pantomime of transitive gestures. Patients with AD scored lower in both subdomains. bvFTD vs. PPA, FTD vs. AD, and PPA vs. AD did not reveal any statistically significant differences. ABA-2, on the other side, revealed distinctive results, with FTD vs. AD having significant differences only in subtest 2 (verbal apraxia), with a higher percentage of pathological findings in the FTD group scoring (85.7% vs. 40.9%). This pattern was also observed when comparing bvFTD vs. AD. Groups of AD vs. PPA differed significantly in subtests 2 and 3B (buccofacial apraxia), with an increased percentage of pathological outcomes in patients with PPA. Finally, the bvFTD and PPA groups had comparable results in all subtests (Table 5).

Subsequently, the correlation between apraxia battery subdomain scores and cognitive decline was investigated. Regarding TULIA, as imitation of transitive actions was deteriorating, the ACE-R score was equally affected (r = 0.555, *p* = 0.007). In the FTD group, similar results were found in all imitation gestures (r = 0.565, *p* < 0.001 for non-symbolic, r = 0.539, *p* < 0.001 for intransitive, and r = 0.520, *p* < 0.001 for transitive movements) as well as pantomiming non-symbolic ones (r = 0.589, *p* < 0.001), whereas in the bvFTD group only when pantomiming non-symbolic movements scores (r = 0.563, *p* < 0.001). Finally, ACE-R showed strong correlation with all movements requiring imitation (r = 0.712, *p* < 0.001; r = 0.751, *p* < 0.001; r = 0.718, *p* < 0.001 for non-symbolic, intransitive, and transitive movements, respectively) and medium when pantomime was applied (r = 0.670, *p* = 0.001; r = 0.554, *p* = 0.007; r = 0.641, *p* = 0.001) in patients with PPA. Further multivariate linear regression analysis revealed no correlation between limb apraxia and cognitive decline in patients with AD. In the bvFTD group, reduction of ACE-R score correlated with lower scores in pantomiming of non-symbolic gestures (R^2^ = 0.348, B = 0.190, *p* = 0.001), while in patients with PPA no correlation was found. Nevertheless, when patients with bvFTD and PPA were analyzed as one group (FTD), cognitive decline was associated with lower scores in all imitation gestures (non-symbolic: R^2^ = 0.383, B = 0.219, *p* < 0.001; intransitive: R^2^ = 0.344, B = 0.144, *p* < 0.001; transitive: R^2^ = 0.337, B = 0.161, *p* < 0.001), as well as in all pantomime gestures (non-symbolic: R^2^ = 0.379, B = 0.241, *p* < 0.001; intransitive: R^2^ = 0.265, B = 0.141, *p* < 0.001; transitive: R^2^ = 0.289, B = 0.127, *p* < 0.001).

Regarding the ABA-2 test, verbal apraxia (subtest 1) and cognitive decline were correlated only in the AD and FTD groups (r = 0.563, *p* = 0.006, and r = 0.479, *p* < 0.001, respectively). All other correlations were not statistically significant. Multivariate linear regression analysis confirmed the correlation of subtest 1 score with cognitive decline in the AD and FTD groups but also demonstrated a similar correlation in the bvFTD group (R^2^ = 0.483, B = 0.375, *p* = 0.011; R^2^ = 0.354, B = 0.274, *p* < 0.001; R^2^ = 0.510, B = 0.282, *p* < 0.001, respectively). Finally, in this analysis, no significant findings were discovered in the PPA group.

## 4. Discussion

Our study indicates the presence of the three major apraxia disturbances in all patients with dementia (AD, bvFTD, and PPA). The sample of patients consisted of individuals with mild or moderate functional disorders, and the performance in both batteries showed difficulties in praxis performance, even at these stages of the disease. Our findings are in accordance with previous studies that evaluated limb and buccofacial apraxia between patients with AD and bvFTD [5,16,17]. All studies showed distinct patterns of apraxia when comparing demented patients with HC, as well as between patient groups. However, in the present study, we also included an assessment of apraxia of speech and extended the evaluation of apraxia in patients with PPA.

Differences in composite scores observed between all subcategories of dementia and HC were statistically significant when both apraxia screening tests were administered. This highlights the role of limb, verbal, and non-verbal apractic disturbances in the clinical setting of AD, bvFTD, and PPA. Hence, integration of all three main praxis tasks in the routine neuropsychological assessment could provide a more complete picture of this cognitive function, expanding former knowledge of specific disabilities in every subgroup [1,18,19].

Composite scores of patients with AD and PPA were lower in the TULIA battery, indicating greater deterioration in limb movements, whereas verbal and non-verbal outcomes (ABA-2) were more often pathological in bvFTD and PPA. Subsequent statistical analyses between overall scores in TULIA showed that individuals diagnosed with AD did not differ significantly when compared with the bvFTD and PPA groups. This reinforces the theory that, in addition to the parietal, the frontal and temporal cortex play an equally important role in limb gestures, and limb apraxia is probably a non-pathognomonic clinical marker for AD but common for these three dementia categories. More specifically, recent literature considers the insula and frontal areas (additionally to the parietal cortex) crucial for the imitation network and the temporal cortex for pantomiming [20]. Interestingly, when comparing limb apraxia between patients with bvFTD and PPA, no statistically significant differences were found. Limb praxis would be expected to be more affected in patients with PPA due to the more widespread pathology, even in the early stages of the disease, but this was not verified. Therefore, the limb praxis profile, using TULIA, cannot serve as a differential diagnosis in cases where the FTD diagnosis is indisputable and its subcategory is under investigation. On the other side, ABA-2 did show significant differences only between FTD and AD groups, suggesting verbal and non-verbal apraxia as a core clinical finding indicating FTD pathology, but was unable to distinguish between bvFTD and PPA cases (the two FTD subtypes, compared with AD, showed increased percentages of pathological outcome but without statistically significant differences).

Limb praxis performance is correlated with global cognitive impairment in patients with FTD and bvFTD. This association was independent of other factors. Generalized and extensive atrophy as well as hypoperfusion result in diminished overall scoring in the TULIA battery. Thus, imitation and pantomime of meaningless, intransitive, and transitive gestures, which are represented in the insula, parietal, frontal, and temporal areas, are affected while the disease is progressing, leading to a gradual disability in the proper execution of mainly demanding but also simple movements. Similar results would be expected in the AD and PPA groups, which did not show a similar correlation, and this may be due to the small sample of patients. Regarding ABA-2 results, a correlation was detected between cognitive decline and verbal/non-verbal apraxia in the FTD population, where the main pathology is located in the frontal lobes and gradual disease progression results in global distortion of speech and face praxis.

All patients scored worse in all subdomains of both tests in comparison with HC, with the results having statistical significance except non-verbal movements (“buccofacial apraxia”—subdomain 3B) and limb apraxia, as it is being roughly examined in the ABA-2 battery (subdomain 3A), between bvFTD and HC. In bvFTD, the presence of limb apraxia, thoroughly examined in the TULIA battery, in addition to verbal apraxia, strengthens recent evidence of the contribution of the frontal brain areas to limb praxis tasks [21]. Likewise, frontal and parietal pathology justify the pathological findings of limb, verbal, and non-verbal apraxia in the PPA group. The AD group was characterized by limb apraxia and, to a lesser degree, by verbal and non-verbal deficits, indicating a milder frontal involvement. These findings highlight the global impairment of the praxis mechanism in all dementia subtypes, regardless of the prevailing area of atrophy. No unique apraxia disturbances were found; however, a heterogeneity in the degree of infestation in the praxis mechanism was observed depending on the diagnosis. Our findings are in line with previous studies suggesting that neuropsychological tests, including assessments of a broad area of apractic disturbances, should be administered when clinically approaching a patient with dementia [22].

The profile of apraxia differed in every patient group when evaluating every subdomain separately. When examining patients with FTD and AD, results from limb praxis execution were comparable, but verbal tasks had significantly increased pathological outcomes in the FTD group. Prominent atrophy in the frontal areas during the course of the disease, in all three main FTD subtypes, results in worse outcomes in verbal apraxia profiles. This clinical finding could serve to confirm a possible diagnosis of the FTD spectrum. When comparing AD and bvFTD groups, imitation of intransitive gestures as well as pantomime of transitive gestures of the dominant hand characterized patients with AD, while verbal apraxia characterized patients with bvFTD. Although the orbitofrontal and prefrontal cortex are considered necessary for facial emotional recognition, the parietal lobe is mainly responsible for imitating intransitive gestures, hence explaining the worse outcome of patients with AD. Additionally, pantomiming transitive gestures requires intact semantic knowledge of tool usage; thus, patients with AD are struggling when an action is verbally asked to be performed [23]. Frontal lobe atrophy, mainly inferior, is correlated with verbal apraxia [24] and can explain the worse outcomes observed in the bvFTD population. Limb apraxia profiles were similar in patients with PPA vs. AD, and this could be explained by the involvement of atrophy in the parietal lobes in both diseases. In contrast, verbal and non-verbal apraxia, caused by frontal lobe atrophy, were prominent in patients with PPA. Surprisingly, despite the multilevel degeneration taking place in the PPA population, results from limb praxis assessments did not differ significantly compared with bvFTD. This highlights the possibly crucial role of the frontal areas in limb praxis. Finally, as expected, verbal and non-verbal apraxia results were comparable between these two patient groups, probably due to frontal area neural damage.

Our final goal was to discover associations between specific apractic deficits and cognitive impairment. A possible correlation could serve as an indirect clinical marker of local functional deterioration, independent of the expected atrophy. As with the composite scores, when approaching each apraxia subdomain, any correlation observed was independent of other factors. Verbal apraxia appeared to be affected by the disease progression in patients with AD. This underlines the possible crucial role of the frontal lobe in functional status deterioration as neurodegeneration accelerates. The expected correlation of limb apraxia (associated with dysfunction of the parietal and temporal lobes) with cognitive decline was left unverified. The small sample of patients participating in this group is suspected to be the main reason for this result. Surprisingly, dysfunction of parietal as well as frontal areas appeared to follow general cognitive impairment in the bvFTD group and led to the corresponding results in verbal apraxia and pantomime of non-symbolic gestures. This raises questions about the importance of parietal functions that get affected by the worsening of the clinical condition of patients with bvFTD. Lastly, none of the apraxia tasks correlated with the ACE-R score in patients with PPA. It can be assumed that the limited number of patients, as with the AD group, and the heterogeneity in their scores contributed to the non-significant results in this group. It is worth mentioning that when assessing verbal apraxia with ABA-2, only subtest 1 (“diadochokinesia”) appeared to be indicative of the ongoing degeneration.

Overall, the presence of a global praxis disturbance in all three patient groups and the distinct findings of apraxia in each subgroup highlight the importance of evaluating apraxia in every-day clinical practice. The treating physician could use this clinical tool when approaching a patient diagnostically. Additionally, taking into consideration that apraxia is correlated with cognitive decline may alter the therapeutic approach and benefit the affected patient population.

### Limitations

Our study incorporated the majority of apractic phenomena experienced by patients with dementia. Results and conclusions emerged from these assessments, but not the entire spectrum of disturbances, which could include, for example, actual use of tools and imitation as well as pantomime of emotional face postures. Research on apraxia in stroke patients has excluded other cognitive dysfunctions, a condition unable to exist in patients with dementia. Memory and visuospatial disturbances are core findings in patients with AD, and their impact on the correct execution of the desired movements is unattainable to estimate. Likewise, verbal comprehension deficits in PPA could play a role in imitating limb and face postures. In the same way, it is unclear to what extent the above cognitive dysfunctions could alter the performance of patients with bvFTD. We can assume that our choice to exclude patients in the severe stage of the disease limits the effect of other cognitive disturbances and points to specific apraxia profiles in all examined patient groups.

We consider as a disadvantage of all published literature the absence of a specific, established, apraxia battery able to assess all aspects of apractic phenomena that could be used by all experts. This leads to the utilization of different tests, depending on the focused deficit, and subsequently to the inability of direct comparison of the results with each other. To overcome this obstacle, we applied two tools focused on different apraxia disturbances, aiming to cover as many aspects of their manifestation as possible. Likewise, this study mainly concerns patients with rare diseases, resulting in limited sample sizes. Compared with the literature, we consider our sample to be satisfactory and acknowledge that only multi-center clinical studies could provide sufficient patient samples. Additionally, as already mentioned, all patients were Greek native speakers, and a modified and standardized version of ABA-2 for the Greek population was administered. Thus, the use of specific language and grammar may pose limitations on the generalizability of the results.

SPECT was used to corroborate the clinical diagnosis of every individual. Thus, areas of hypoperfusion were identified and linked to clinical expressions. Findings from SPECT cannot reveal neural pathways but only regional impairments, giving a rough estimate of focal dysfunction, which could possibly be the pathogenic cause or only a part of the malfunctioning system. Our study was focused on revealing apraxia profiles capable of distinguishing AD, bvFTD, and PPA from each other on a clinical level, leaving the area of pathogenetic mechanisms unexplored.

## 5. Conclusions

In this research, we found that patients with AD, bvFTD, and PPA suffer from specific apractic disturbances. Both apraxia tools (TULIA and ABA-2) were used in the differential diagnostic process between dementia groups. Limb praxis deficits were prominent in the PPA and AD groups, whereas verbal and non-verbal apraxia indicated FTD pathology. When summarizing all the results, patients with AD were distinguished by significantly worse performance in imitating intransitive gestures as well as pantomiming transitive gestures when compared with bvFTD, with the latter group being characterized by verbal apraxia. AD vs. PPA groups differed only in verbal and non-verbal apraxia, which was prominent in the PPA group. Finally, limb, verbal, and non-verbal praxis assessments were unable to distinguish patients with PPA from bvFTD, but the former group suffers from greater deterioration of both limb and non-verbal praxis. This could be used as an indicative, but not diagnostic, clinical marker. Additionally, certain praxis tasks are correlated with cognitive decline, indicating corresponding neurodegeneration as the disease progresses. Considering that the diagnostic value of biomarkers is under investigation and, especially for patients with FTD, reliable laboratory indicators are being explored, characteristics in the clinical picture of patients still play a major role in the differential diagnosis process. Based on these insights, detailed apraxia screening could contribute to a more comprehensive clinical evaluation, aiding in the differentiation of dementia subtypes. Further studies exploring all aspects of apraxia and the correlation between structural and functional lesions and specific apraxia patterns would provide further information about its clinical validity and neural basis. This could lead to a better understanding of the degenerative progress, the manifestations of the clinical spectrum, the diagnostic algorithm that could be followed, and a possible therapeutic approach for each of these dementia syndromes.

## Figures and Tables

**Table 1:** Patient characteristics and neuropsychological/apraxia assessment.

	AD (n = 22)	FTD (n = 63)	bvFTD (n = 41)	PPA (n = 22)	HC (n = 20)
Demographics					
Age * (years)	69.71 ± 6.15	68.38 ± 9.13	68.68 ± 9.98	67.05 ± 7.31	69.21 ± 6.51
Sex ** (m/f)	12/10	36/27	25/16	11/11	11/13
Disease duration *** (y)	3.05 ± 2.01	3.27 ± 2.13	3.35 ± 2.21	3.10 ± 1.99	0.08 ± 0.41
Education *** (y)	10.24 ± 4.22	9.57 ± 4.55	9.07 ± 4.86	10.18 ± 3.80	11.63 ± 3.83
Neuropsychological/apraxia assessment					
ACE-R *	63.05 ± 14.72	56.86 ± 23.56	60.05 ± 20.94	50.91 ± 27.33	96.17 ± 3.41
FRS *	2.11 ± 1.50	1.37 ± 1.80	1.33 ± 1.72	1.44 ± 1.99	4.86 ± 0.74
FBI *	4.95 ± 4.37	14.25 ± 8.48	16.34 ± 7.53	10.36 ± 8.95	2.58 ± 2.69
TULIA *	188.45 ± 12.82	190.37 ± 41.57	198.90 ± 27.13	174.45 ± 57.38	228.79 ± 6.59
ABA-2 **	63.6	90.5	90.2	90.9	4.2

* Data represent mean ± standard deviation. ** Data represent percentage of patients with pathological outcome. *** Data represent median (interquartile range). HC: Health Control, AD: Alzheimer’s Disease, FTD: Frontotemporal Dementia, bvFTD: behavioral variant FTD, PPA: Primary Progressive Aphasia, ACE-R: Adenbrooke’s Cognitive Examination—Revised, FRS: Frontal Rating Scale, FBI: Frontal Behavioral Inventory, TULIA: Test for Upper Limb Apraxia, ABA-2: Apraxia Battery for Adults—2.

**Table 2:** Between-group comparisons of composite scores in TULIA and ABA-2.

	HC vs. Dementia Groups				Comparisons between Dementia Groups			
	HC vs. AD	HC vs. FTD	HC vs. bvFTD	HC vs. PPA	FTD vs. AD	bvFTD vs. AD	bvFTD vs. PPA	AD vs. PPA
TULIA ^#^	40.34 ***	38.42 ***	29.89 ***	54.34 ***	1.92	10.45	24.45	14.00
ABA-2 ^†^	−59.4 ***	−86.3 ***	−86.0 ***	−86.7 ***	26.9 *	26.6 *	−0.7	−27.3

^#^ Data represent mean score difference in TULIA battery. ^†^ Data represent differences in percentage of patients with pathological outcome in ABA-2 battery. Statistically significant findings shown with bold. * *p*-value < 0.05, *** *p*-value < 0.001. HC: Health Control, AD: Alzheimer’s Disease, FTD: Frontotemporal Dementia, bvFTD: behavioral variant FTD, PPA: Primary Progressive Aphasia, TULIA: Test for Upper Limb Apraxia, ABA-2: Apraxia Battery for Adults—2.

**Table 3:** Sensitivity and specificity properties for each dementia group.

		Total Patients vs. HC	AD vs. HC	FTD vs. HC	bvFTD vs. HC	PPA vs. HC
TULIA	AUC (CI 95%)	0.911 (0.859–0.963)	1.000 (1.000–1.000)	0.880 (0.811–0.949)	0.877 (0.794–0.960)	0.885 (0.778–0.993)
	Cut-off Score	220.50	215.00	220.50	223.50	220.50
	Sensitivity (%)	83.5	100	77.8	82.9	81.8
	Specificity (%)	87.5	100	87.5	79.2	87.5
ABA-2	Sensitivity (%)	83.5	63.6	90.4	90.2	90.9
	Specificity (%)	95.8	95.8	95.8	95.8	95.8

HC: Health Control, AD: Alzheimer’s Disease, FTD: Frontotemporal Dementia, bvFTD: behavioral variant FTD, PPA: Primary Progressive Aphasia, TULIA: Test for Upper Limb Apraxia, ABA-2: Apraxia Battery for Adults—2, AUC: Area Under Curve.

**Table 4:** TULIA and ABA-2 subdomains scores and comparison with HC.

			HC	AD	FTD	bvFTD	PPA	HC vs. AD	HC vs. FTD	HC vs. bvFTD	HC vs. PPA
TULIA ^#^	Imitation	Non-symbolic	37.30 ± 1.49	26.45 ± 5.18	28.32 ± 8.43	29.66 ± 6.42	25.82 ± 11.02	10.84 ***	8.97 ***	7.63 ***	11.47 ***
		Intransitive	38.80 ± 1.15	32.36 ± 2.08	33.11 ± 6.69	34.46 ± 4.46	30.59 ± 9.17	6.39 ***	5.64 ***	4.29 ***	8.16 ***
		Transitive	38.60 ± 1.50	34.32 ± 3.67	32.86 ± 7.41	34.41 ± 4.56	29.95 ± 10.45	4.31 ***	5.77 ***	4.22 ***	8.68 ***
	Pantomime	Non-symbolic	37.30 ± 1.67	28.68 ± 3.36	28.86 ± 8.98	30.27 ± 7.25	26.23 ± 11.27	8.57 ***	8.39 ***	6.98 ***	11.02 ***
		Intransitive	39.40 ± 1.06	34.68 ± 3.68	33.52 ± 7.66	35.05 ± 5.02	30.68 ± 10.60	4.70 ***	5.86 ***	4.33 ***	8.70 ***
		Transitive	37.60 ± 1.82	31.95 ± 3.11	33.71 ± 6.73	35.07 ± 3.95	31.18 ± 9.67	5.63 ***	3.87 **	2.51 **	6.40 **
ABA-2 ^†^		Diadochokinesia	0	40.9	58.7	53.7	68.2	40.9 ***	58.7 ***	53.7 ***	68.2 ***
		Increasing Word Length	4.2	40.9	85.7	85.4	86.4	36.7 *	81.5 ***	81.2 ***	82.2 ***
		Limb Apraxia	0	31.8	27.0	24.4	31.8	31.8 *	27.0 *	24.4 *	31.8 *
		Buccofacial Apraxia	0	0	19.0	12.2	31.8	-	19.0 *	12.2	31.8 *
		Latency Time and Utterance Time for Polysyllabic Words	0	9.1	11.1	9.8	13.6	9.1	11.1	9.8	13.6

^#^ TULIA scores are shown as mean ± standard deviation and between-group comparison data as mean score differences. ^†^ ABA-2 results are shown as percentage of patients with pathological outcome and between-group comparison data as differences in percentages of pathological outcome. Statistically significant findings shown with bold. * *p*-value < 0.05, ** *p*-value < 0.01, *** *p*-value < 0.001 HC: Healthy control, AD: Alzheimer’s disease, FTD: Frontotemporal dementia, bvFTD: behavioral variant FTD, PPA: Primary progressive aphasia, TULIA: Test for Upper Limb Apraxia, ABA-2: Apraxia Battery for Adults—2.

**Table 5:** Comparison of TULIA and ABA-2 results between dementia groups.

TULIA ^#^		**Imitation**			**Pantomime**		
		**Non-Symbolic**	**Intransitive**	**Transitive**	**Non-Symbolic**	**Intransitive**	**Transitive**
	FTD vs. AD	1.87	0.75	−1.46	0.18	−1.16	1.76
	bvFTD vs. AD	3.21	2.10 *	0.09	1.59	0.37	3.12 *
	bvFTD vs. PPA	3.84	3.87	4.46	4.04	4.37	3.89
	AD vs. PPA	0.63	1.77	4.37	2.45	4.00	0.77
ABA-2 ^†^		Diadochokinesia	Increasing Word Length	Limb Apraxia	Buccofacial Apraxia	Latency Time and Utterance Time for Polysyllabic Words	
	FTD vs. AD	17.8	44.8 ***	−4.8	19.0	2.0	
	bvFTD vs. AD	12.8	44.5 **	−7.4	12.2	0.7	
	bvFTD vs. PPA	−14.5	−1.0	−7.4	−19.6	−3.8	
	AD vs. PPA	−27.3	−45.5 **	0	−31.8 *	−4.5	

^#^ Data represent mean score difference in TULIA battery. ^†^ Data represent differences in percentage of patients with pathological outcome in ABA-2 battery findings shown with bold. * *p*-value < 0.05, ** *p*-value < 0.01, *** *p*-value < 0.001. HC: Health Control, AD: Alzheimer’s Disease, FTD: Frontotemporal Dementia, bvFTD: behavioral variant FTD, PPA: Primary Progressive Aphasia, TULIA: Test for Upper Limb Apraxia, ABA-2: Apraxia Battery for Adults—2.

## Data Availability

The data presented in this study are available on request from the corresponding author.

## References

[1] McKhann G.M., Knopman D.S., Chertkow H., Hyman B.T., Jack C.R., Kawas C.H., Klunk W.E., Koroshetz W.J., Manly J.J., Mayeux R. (2011). The diagnosis of dementia due to Alzheimer’s disease: Recommendations from the National Institute on Aging-Alzheimer’s Association workgroups on diagnostic guidelines for Alzheimer’s disease. Alzheimer’s Dement..

[2] Gorno-Tempini M.L., Hillis A.E., Weintraub S., Kertesz A., Mendez M., Cappa S.F., Ogar J.M., Rohrer J.D., Black S., Boeve B.F. (2011). Classification of primary progressive aphasia and its variants. Neurology.

[3] Coslett H.B. (2018). Apraxia, Neglect, and Agnosia. Contin. Lifelong Learn. Neurol..

[4] Roy E.A., Elliot D. (1996). Hand Preference, Manual Assymetries, and Limb Apraxia. Manual Asymmetries in Motor Performance.

[5] Johnen A., Tokaj A., Kirschner A., Wiendl H., Lueg G., Duning T., Lohmann H. (2015). Apraxia profile differentiates behavioural variant frontotemporal from Alzheimer’s dementia in mild disease stages. J. Neurol. Neurosurg. Psychiatry.

[6] Rascovsky K., Hodges J.R., Knopman D., Mendez M.F., Kramer J.H., Neuhaus J., Van Swieten J.C., Seelaar H., Dopper E.G., Onyike C.U. (2011). Sensitivity of revised diagnostic criteria for the behavioural variant of frontotemporal dementia. Brain.

[7] Konstantinopoulou E., Kosmidis M.H., Ioannidis P., Kiosseoglou G., Karacostas D., Taskos N. (2011). Adaptation of Addenbrooke’s Cognitive Examination-Revised for the Greek population. Eur. J. Neurol..

[8] Konstantinopoulou E., Aretouli E., Ioannidis P., Karacostas D., Kosmidis M.H. (2013). Behavioral disturbances differentiate frontotemporal lobar degeneration subtypes and Alzheimer’s disease: Evidence from the Frontal Behavioral Inventory. Int. J. Geriatr. Psychiatry.

[9] Mioshi E., Hsieh S., Savage S., Hornberger M., Hodges J.R. (2010). Clinical staging and disease progression in frontotemporal dementia. Neurology.

[10] Valotassiou V., Malamitsi J., Papatriantafyllou J., Dardiotis E., Tsougos I., Psimadas D., Alexiou S., Hadjigeorgiou G., Georgoulias P. (2018). SPECT and PET imaging in Alzheimer’s disease. Ann. Nucl. Med..

[11] Archer H.A., Smailagic N., John C., Holmes R.B., Takwoingi Y., Coulthard E.J., Cullum S. (2015). Regional Cerebral Blood Flow Single Photon Emission Computed Tomography for detection of Frontotemporal dementia in people with suspected dementia (Review). Cohrane Libr. Cohrane Database Syst. Rev..

[12] Bossuyt P.M., Reitsma J.B., Bruns D.E., Gatsonis C.A., Glasziou P.P., Irwig L., Lijmer J.G., Moher D., Rennie D., De Vet H.C. (2015). STARD 2015: An updated list of essential items for reporting diagnostic accuracy studies. Radiology.

[13] Vanbellingen T., Kersten B., Van Hemelrijk B., Van De Winckel A., Bertschi M., Müri R., De Weerdt W., Bohlhalter S. (2010). Comprehensive assessment of gesture production: A new test of upper limb apraxia (TULIA). Eur. J. Neurol..

[14] Tafiadis T., Keloglou M., Zafeiri A.T.M., Tafiadis D., Keloglou M., Zafeiri A., Tafiadi M. (2010). The Apraxia battery for Adults—2 (ABA-2). (A second pilot study and validation of the test in aphasic Greek population). Ann. Gen. Psychiatry.

[15] Papadopoulos G., Parissis D., Konstantinopoulou E., Natsis K., Gotzamani-Psarrakou A., Ioannidis P. (2021). Preliminary validation of the apraxia battery for adults-second edition (ABA-2) in Greek patients with dementia. Acta Neurol. Belg..

[16] Johnen A., Reul S., Wiendl H., Meuth S.G., Duning T. (2018). Apraxia profiles—A single cognitive marker to discriminate all variants of frontotemporal lobar degeneration and Alzheimer’ s disease. Alzheimer’s Dement..

[17] Yliranta A., Jehkonen M. (2020). Limb and face apraxias in frontotemporal dementia: A systematic scoping review. Cortex.

[18] Silveri M.C., Pravatà E., Brita A.C., Improta E., Ciccarelli N., Rossi P., Colosimo C. (2014). Primary progressive aphasia: Linguistic patterns and clinical variants. Brain Lang..

[19] Chandra S., Issac T., Abbas M. (2015). Apraxias in neurodegenerative dementias. Indian J. Psychol. Med..

[20] Lesourd M., Francois O., Baumard J., Bartolo A., Vanbellingen T., Reynaud E. (2018). Cerebral correlates of Imitation of Intransitive Gestures: An Integrative Review of Neuroimaging Data and Brain Lesion Studies. Neurosci. Biobehav. Rev..

[21] Johnson-Frey S.H. (2004). The neural bases of complex tool use in humans. Trends Cogn. Sci..

[22] Johnen A., Frommeyer J., Modes F., Wiendl H., Duning T., Lohmann H. (2015). Dementia Apraxia Test (DATE): A brief tool to differentiate behavioral variant frontotemporal dementia from Alzheimer’s dementia based on apraxia profiles. J. Alzheimer’s Dis..

[23] Cotelli M., Manenti R., Brambilla M., Balconi M. (2014). Limb apraxia and verb processing in Alzheimer’s disease. J. Clin. Exp. Neuropsychol..

[24] Ballard K.J., Tourville J.A., Robin D.A. (2014). Behavioral, computational, and neuroimaging studies of acquired apraxia of speech. Front. Hum. Neurosci..

